# Epithelioid hemangioendothelioma of the thyroid: a case report

**DOI:** 10.1186/s40792-017-0294-2

**Published:** 2017-01-28

**Authors:** Mayu Ono, Yoshio Kasuga, Takeshi Uehara, Yoshinao Oda

**Affiliations:** 1 Department of Breast and Endocrine Surgery, Nagano Matsushiro General Hospital, 183 Matsushiro, Nagano, 381-1231 Japan; 2 Department of Laboratory Medicine, Shinshu University School of Medicine, 3-1-1 Asahi, Matsumoto, 390-8621 Japan; 3 Department of Anatomic Pathology, Pathological Sciences, Graduate School of Medical Sciences, Kyushu University, 3-1-1 Maidashi, Higashi-ku, Fukuoka 812-8582 Japan; 4 Present address: Department of Breast and Endocrine Surgery, Iida Municipal Hospital, 438 Yawatamachi, Iida, 812-8582 Japan

**Keywords:** Epithelioid hemangioendothelioma, Thyroid, Surgery

## Abstract

**Background:**

Epithelioid hemangioendothelioma (EHE) of the thyroid is an extremely rare disease; only three cases have been reported in the English literature to date. Here, we describe a case involving a patient with thyroid EHE successfully treated with curative surgery.

**Case presentation:**

A 74-year-old woman presented with a right thyroid mass. The nodule was approximately 2 cm in size and was diagnosed as an indeterminate lesion by fine needle aspiration cytology. She was treated with thyroid lobectomy. The histopathological and immunohistochemical findings indicated an EHE of the thyroid. At the latest follow-up, 3 years postoperatively, the patient showed no signs of recurrence.

**Conclusion:**

There is currently no standard therapy for EHE; however, our case suggests that curative resection represents an effective treatment.

## Background

Epithelioid hemangioendothelioma (EHE) is a rare vascular tumor, most commonly reported to occur in the lungs, liver, and bone, along with many other sites throughout the body [1]. The etiology of EHE is unknown. Previously, EHE was recognized as an intermediate-risk disease, classified between angioma and angiosarcoma [2]; however, in the fourth revision of the World Health Organization classification, EHE was described as a malignant vascular tumor, similar to angiosarcoma [3].

EHE of the thyroid is very rare, and only three cases have been reported to date in the English literature. Here, we describe a patient with EHE of the thyroid who underwent successful resection.

## Case presentation

**Fig. 1:**
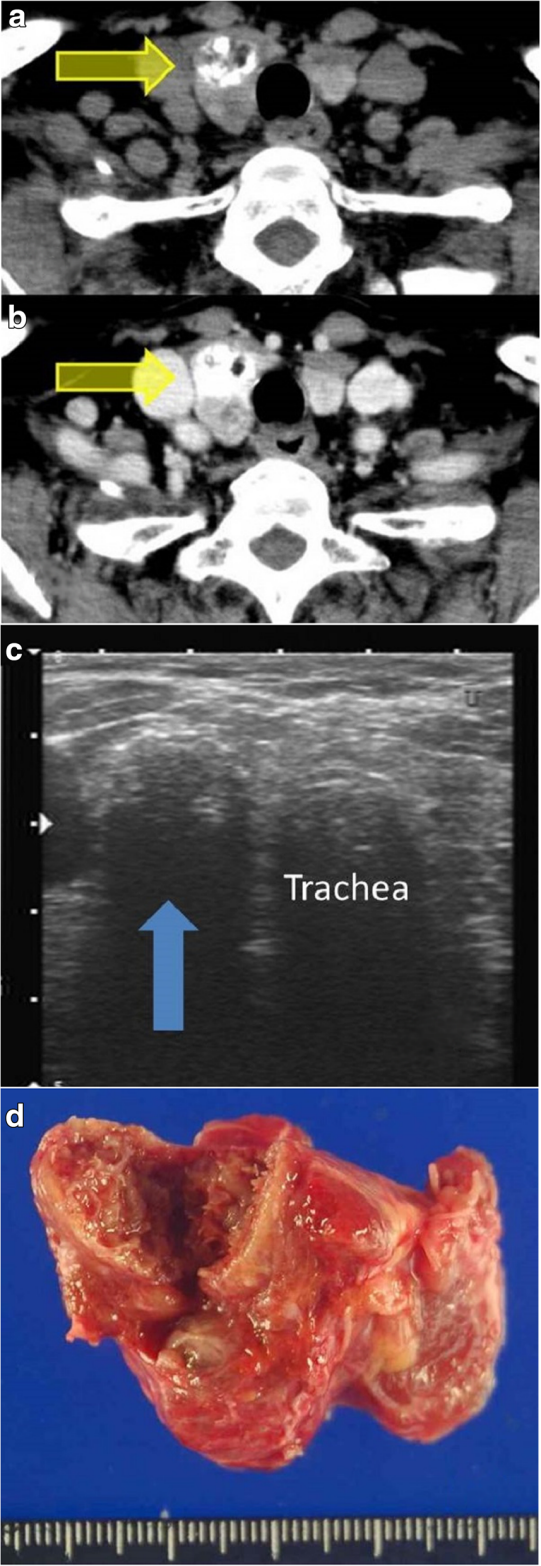
Characteristics of the thyroid nodule. Computed tomography scan showing the right thyroid nodule with calcification (*arrow*) (**a**). The nodule is enhanced and shows no signs of extrathyroidal invasion (*arrow*) (**b**). Ultrasonogram showing the right thyroid nodule (21 mm in diameter) with calcification (*arrow*) (**c**). Macroscopically, a well-circumscribed mass in the right lobe of the thyroid is observed (**d**)

**Fig. 2:**
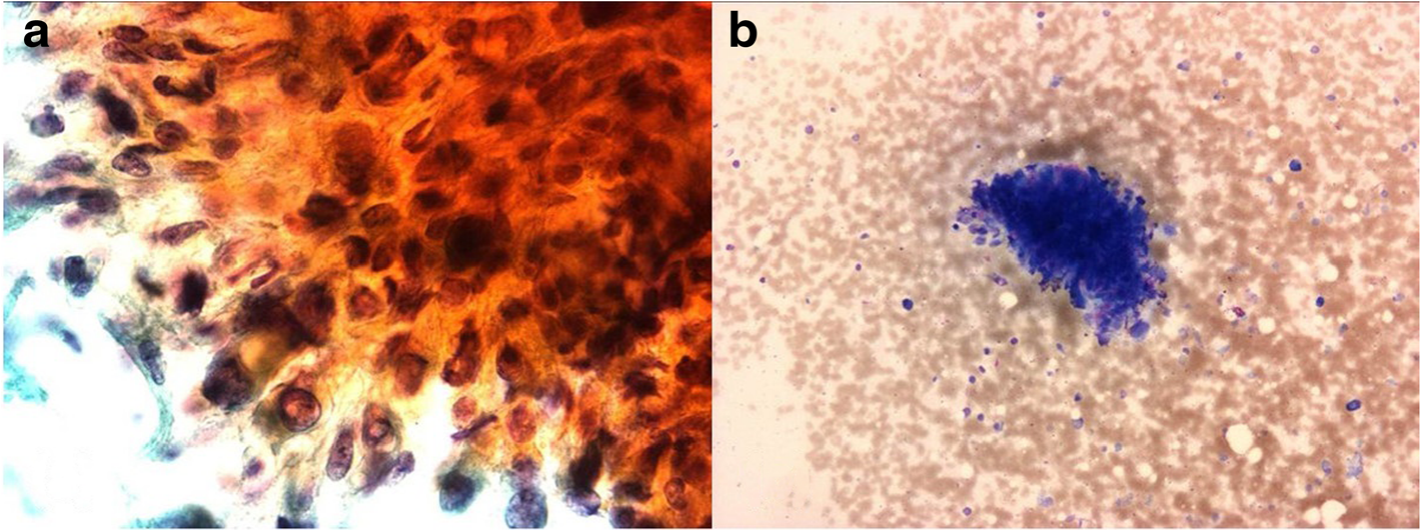
Fine needle aspiration smears. Spindle-shaped cells are observed, with prominent nucleoli and nuclear inclusion (Papanicolaou stain, ×400) (**a**). The intercellular space is metachromatic (Giemsa stain, ×100) (**b**)

A 74-year-old woman was referred to our hospital because of a nodule in the right lobe of the thyroid. The nodule had been found during a medical examination performed 3 years ago, but she did not undergo a detailed examination at the local hospital at that time. On physical examination, a palpable and hard nodule of approximately 2 cm in size was noted in the right side of her neck. Results of the blood examination were normal, except for mildly increased thyroglobulin (142 ng/mL) and antithyroglobulin antibody levels (59.7 IU/mL). Ultrasonography and computed tomography showed a right thyroid nodule with calcification. The nodule had no signs of extrathyroidal invasion and measured 21 mm in diameter. The lymph nodes of the neck were not swollen (Fig. 1). The nodule was diagnosed as an indeterminate lesion by fine needle aspiration cytology. The fine needle aspiration smears showed spindle-shaped cells with prominent nucleoli and nuclear inclusion. The intercellular space was metachromatic on Giemsa staining (Fig. 2). Taken together, these findings suggested a hyalinizing trabecular adenoma, granulomatous lesion, papillary carcinoma, or poorly differentiated carcinoma.

**Fig. 3:**
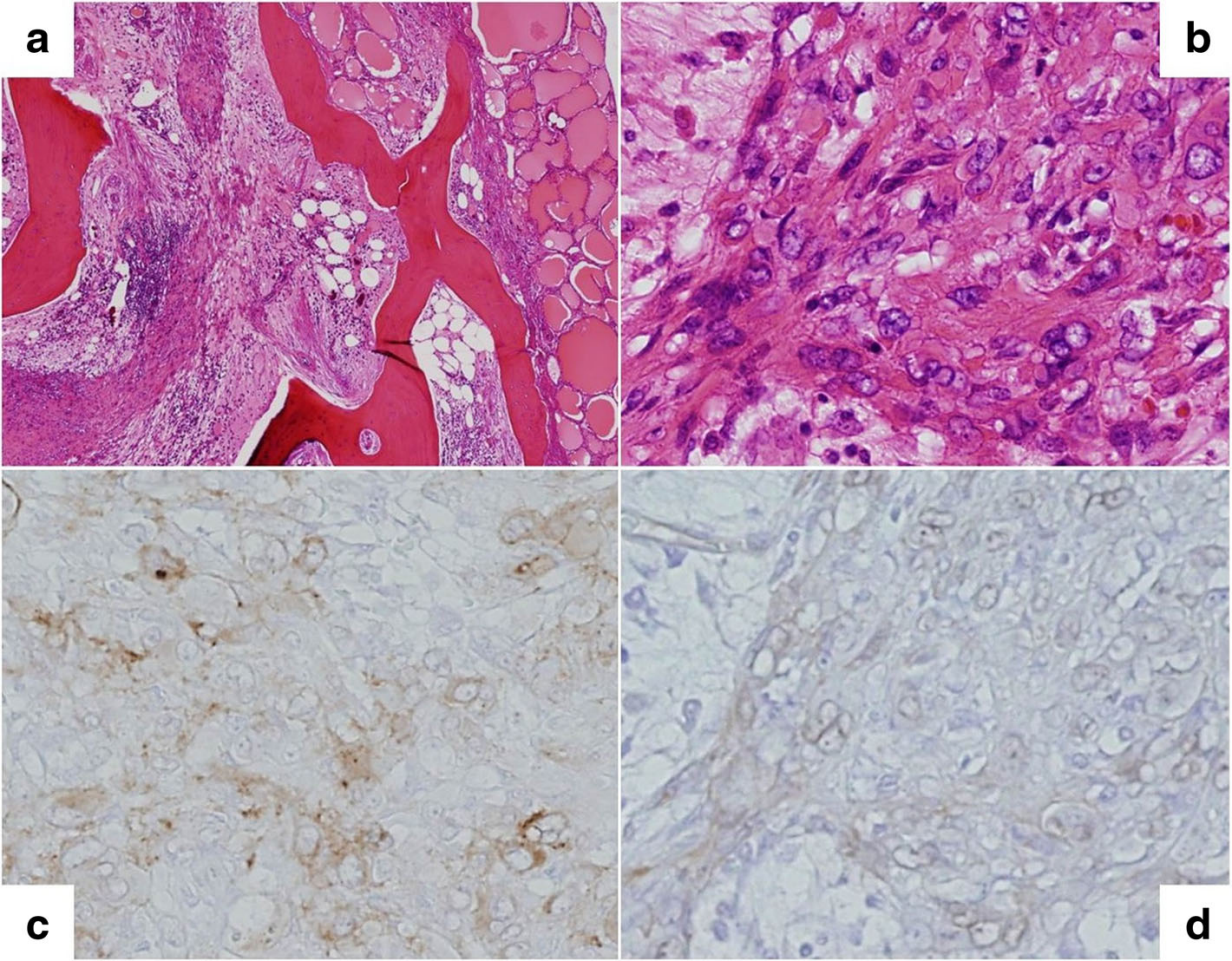
Histological findings. Multinodular proliferations, composed of comparatively rich spindle cells with nuclear blebs, are observed between the trabecular bone structures. Intracytoplasmic vacuoles are also seen (**a** hematoxylin and eosin stain, ×10; **b** hematoxylin and eosin stain, ×100). Immunohistochemically, the tumor cells were positive for cluster of differentiation (CD)34 and factor VIII (**c** factor VIII, ×100; **d** CD34, ×100)

We decided to treat this nodule as thyroid cancer. Right thyroid lobectomy and central neck dissection were performed. Macroscopically, a well-circumscribed mass in the right lobe of the thyroid was observed. Histologic evaluation of the thyroid nodule showed multinodular proliferation of oval to polygonal-shaped epithelioid cells with oval nuclei and eosinophilic cytoplasm arranged in sheet- or cord-like patterns, accompanied by fibrous stroma and marked osseous metaplasia. Nuclear atypia was mild, and mitosis was not prominent. Results of the immunohistochemical staining showed that these areas were partially positive for cytokeratin, cluster of differentiation (CD)34, and factor VIII; diffusely positive for vimentin; and negative for thyroglobulin, thyroid transcription factor-1, smooth muscle actin, desmin, S100, CD31, CD68, and CD163 (Fig. 3). These findings were suggestive of EHE.

Postoperatively, the patient’s thyroglobulin level fluctuated inconsistently (range, 11–292 ng/mL), as did the antithyroglobulin antibody level, although it remained positive. The mild elevation of the preoperative and postoperative thyroglobulin levels might have been caused by chronic thyroiditis. The patient has been followed-up for 3 years and has had no signs of recurrence postoperatively.

## Conclusions

EHE is a rare disease, first described by Weiss et al. in 1982 [2]. The authors classified it as a vascular tumor, showing the same histological findings, but reported it as a different disease in each organ (e.g., intravascular bronchioloalveolar tumor of the lung) [4]. The 2002 World Health Organization classification changed the classification of EHE from an intermediate to malignant tumor, because some malignant clinical cases had been reported in patients with EHE. EHE most commonly presents in the liver, lung, and bone [5]. The characteristics of EHE in these common sites are shown in Table 1. In addition, Table 2 summarizes the reported EHE cases from various, rare sites of the body.

**Table 1:** Characteristics of epithelioid hemangioendothelioma (EHE) in the liver, lungs, soft tissues, and bone (common presentations)

Site [ref]	Sex	Multicentric	Metastasis		Management	Survival rate (5 years)	Prognostic factors	Treatment
			Rate (%)	Site				
Liver [14–19]	M < F	87%	36.6	Lungs, peritoneum, lymph nodes, bone	Transplantation, resection, medication, radiotherapy, embolization	41	Extrahepatic disease, vascular invasion	Chemotherapy (doxorubicin, cisplatin, 5FU), targeted therapy (sorafenib, pazopanib), IFN α-2b, thalidomide, lenalidomide
Lung [1, 20–23]	M < F	91%	50.5	Liver, pleura, lymph nodes	Lung resection, medication	60	Hemoptysis, pleural effusion, anemia, thoracic symptoms	Chemotherapy (carboplatin, paclitaxel), targeted therapy (bevacizumab, pazopanib), IFN α-2b, thalidomide, lenalidomide
Soft tissue [10, 24]	M < F	4%	22	Lungs, lymph nodes, liver, bone	Surgical resection, chemotherapy, radiotherapy	81	Mitotic activity, size	ND
Bone [25–27]	M > F	>50%	ND	Uncommon	Wide surgical resection, limited surgery, radiofrequency ablation, radiotherapy	92 (10 years)	Visceral involvement	

*M* male, *F* female, *5FU* 5-fluorouracil, *IFN* interferon, *ND* not determined

**Table 2:** Summary of previously reported cases of epithelioid hemangioendothelioma (EHE) occurring in rare sites

Author [ref]	Site	Treatment	Metastasis/reccurence	Outcome (follow-up period)
Barger [28]	Brain	Resection(this case), chemotherapy, radiotherapy, gamma knife, sunitinib	(−)/ND	Alive (1 month)
Sancheti [29]	Hypopharynx	Excision	(−)/(−)	Alive (1 year)
Boscaino [30]	Larynx	Resection	ND/ND	ND
Pigadas [31]	Parotid salivary gland	Parotidectomy	(−)/(−)	Alive (18 months)
Moulai [32]	Heart	Chemotherapy, cardiac transplantation	(−)/(+) 2 years	Alive (10 years)
Traverse [33]	Aorta	Resection	(−)/ND	ND
Charette [34]	Vein	Resection	(−)/(−)	Alive (18 months)
Versaci [35]	Stomach	Resection	(−)/(−)	Alive (8 months)
Ratan [36]	Greater omentum	Resection	(−)/(−)	Alive (6 years)
Bozkurt [37]	Adrenal gland	Laparoscopic excision	(−)/(−)	Alive (6 months)
Tolkach [38]	Kidney	Nephrectomy, sunitinib	(−)/(+) 2 m	Dead (33 months)
Liu [39]	Bladder	Transurethral resection	(−)/(−)	Alive (6 months)
Elhosseiny [40]	Penis	Local excision	(−)/(−)	Alive (ND)
Illueca [41]	Ovary	Resection	(−)/(−)	Alive (1 year)

Especially, EHE of the thyroid is extremely rare [6–9], with only one case reported in the Japanese literature [9], along with three in the English literature [6–8]. These cases and our present case are summarized in Table 3.

**Table 3:** Cases of thyroid epithelioid hemangioendothelioma (EHE)

Author/year [ref]	Age (years)	Sex	Thyroid tumor		Surgical procedure		Metastasis	Adjuvant therapy	Recurrence	Outcome (follow-up period)
			Location	Size (mm)	Thyroid	Neck dissection				
Fujiwara/1998 [9]	56	M	Left lobe	30	Lobectomy	Left lateral	Lymph node	Radiation	(−)	Alive (11 months)
Siddiqui/1998 [6]	44	F	Right lobe	37	Lobectomy	Unknown	Unknown	(−)	(−)	Alive (24 months)
Hassan/2005 [7]	73	F	Right lobe	80	Total thyroidectomy	Unknown	Unknown	IFN-α	(+) 9 months	Dead (13 months)
Shah/2016 [8]	35	F	Left lobe	27	Total thyroidectomy	Left lateral	Lymph node	Plans for radiation and chemotherapy	(−)	Alive (4 months)
Our case/2016	74	F	Right lobe	21	Lobectomy	Central	(−)	(−)	(−)	Alive (36 months)

*M* male, *F* female

Of note, only one of the five patients was male (20%), which is in accordance with previous studies on EHE of other organs, in which most patients were women (male to female ratio, 1:4) [1]. There was no predominance in age (range, 35–74 years) or tumor size (range 2.1–8 cm), but all patients had a single nodule of the thyroid. On the other hand, EHE of other organs associated with multiple organ involvement in 36% of cases [5].

There have been several discussions on the prognostic factors of EHE, such as the presence of pulmonary lesions, multiorgan involvement, age, and sex [1]. In addition, mitotic activity (>3 mitotic figures/50 high-power fields) and size (>3.0 cm) have been reported as risk factors for mortality in cases of EHE of the soft tissues [10]. Although the thyroid EHE patient with the largest tumor (80 mm) died 13 months after the diagnosis [7], the other patients, whose tumor sizes were all <4 cm, had uneventful outcomes [6, 8, 9].

There is no standard treatment for EHE, although curative resection has been reported as a successful treatment with good outcomes [1]. All five patients with thyroid EHE underwent surgery. For this site, total resection can be relatively easily performed, whereas this treatment is unlikely to be performed for EHEs of other organs such as the liver, bone, and lungs. However, it is difficult to maintain a sufficient surgical margin in cases of large thyroid tumors. Our patient had the smallest tumor of the five cases, and no signs of adhesion, extrathyroidal extension, and lymph node metastasis. As mentioned above, the previously reported patient with the largest tumor experienced local recurrence and died. That tumor showed adhesion, whereas pathologically, there was no evidence of a residual tumor [7]. These findings suggest that tumor size is likely an important factor for the effectiveness of curative resection for thyroid EHE. Further studies are needed to determine the optimal surgical margin and whether lymph node dissection is sufficient for curative surgery. Moreover, two patients received radiation therapy because of lymph node metastasis. Radiation therapy may be effective to control local recurrences; however, this could not be confirmed, because the follow-up periods in the past cases were short.

Finally, the presence of CAMTA1-WWTR1 gene fusion in EHE was recently demonstrated [11]. This abnormality has been reported in the majority of EHEs at various anatomical sites, while it is absent in other epithelioid vascular tumors [11–13]. It was also confirmed in one case of thyroid EHE [8]. Therefore, fluorescence in situ hybridization analysis to detect CAMTA-WWTR1 fusion may become a definite tool for the diagnosis of EHE in the future.

In conclusion, EHE of the thyroid is an extremely rare disease. Its clinical course varies, and the prognostic factors are unclear. Curative resection may be an effective treatment, but surgical issues such as the appropriate extent of the surgical margin and use of lymph node dissection remain to be clarified. Further studies are needed to analyze the etiology of EHE and to determine the optimal treatment.

## Abbreviations

CD: Cluster of differentiation
EHE: Epithelioid hemangioendothelioma
